# A Prospective Multi-Center Newborn Screening for Thalassemia by Comprehensive Analysis of Thalassemia Alleles (CATSA) Based on Single Molecule Real-Time Sequencing in Guangxi, China

**DOI:** 10.3390/ijns12020037

**Published:** 2026-05-22

**Authors:** Aihua Xia, Hongfei Chen, Fuhua Lu, Ping Xu, Peixiao Shen, Wei Wei, Chunrong Gui, Juliang Liu, Dan Wei, Haipeng Qin, Yan Huang, Ju Long, Baoheng Gui

**Affiliations:** 1 Obstetrics and Gynecology, The Ninth Affiliated Hospital of Guangxi Medical University, Beihai 536006, China; xiaaihua1004@163.com (A.X.); zjinrong@163.com (P.X.); 2 The Second School of Medicine, Guangxi Medical University, Nanning 530007, China; chenhongfei@gxmu.edu.cn; 3 Obstetrics and Gynecology, Qinzhou Maternal and Child Health Care Hospital, Qinzhou 535099, China; lufuhua2023@163.com (F.L.); spx2100@163.com (P.S.); 4 Center for Medical Genetics and Genomics, The Second Affiliated Hospital of Guangxi Medical University, Nanning 530007, China; 18275746212@163.com (W.W.); guichunrong525@163.com (C.G.); 15278001639@163.com (J.L.); danwei419@163.com (D.W.); humphrey_apengs@163.com (H.Q.); 5 The Guangxi Health Commission Key Laboratory of Medical Genetics and Genomics, The Second Affiliated Hospital of Guangxi Medical University, Nanning 530007, China; 6 Department of Obstetrics, The Second Affiliated Hospital of Guangxi Medical University, Nanning 530007, China; 7 Laboratory of Medical Genetics, Qinzhou Maternal and Child Health Care Hospital, Qinzhou 535099, China

**Keywords:** thalassemia, prevention and control, newborn screening, SMRT, prospective study, multi-center

## Abstract

Thalassemia is one of the most common inherited diseases in Guangxi, China. Early identification of thalassemia by neonatal screening is beneficial for effective clinical management and treatment. A total of 3671 newborns from multiple centers of Guangxi were prospectively recruited and screened for thalassemia using single molecule real-time (SMRT) sequencing technology. A total of 36 types of variants of globin genes were identified, including 16 common variants and 20 rare variants in the Chinese population. In total, 956 (26.04%) newborns were identified to carry thalassemia variants, including 672 (18.31%) α-thalassemia, 228 (6.21%) β-thalassemia, 55 (1.50%) combined α/β-thalassemia and 1 (0.03%) δ-thalassemia. In addition, this study showed that the carrier rates of structural variants of α-globin genes and abnormal hemoglobin variants were 1.28% and 0.93% respectively. Phenotypically, 12 newborns with hemoglobin H disease and 2 cases with intermedia β-thalassemia were found, two of whom would be misdiagnosed by conventional genetic analysis methods. Collectively, this study characterized the complexity and diversity of thalassemia gene variants in newborns of Guangxi, and further achieved early identification of newborns with intermedia thalassemia, which facilitated precision prevention of thalassemia in this region. Also, SMRT provided a powerful tool for neonatal thalassemia screening, especially in prevalent regions.

## 1. Introduction

Thalassemia is a group of autosomal recessive hemoglobinopathies resulting from reduced or absent synthesis of globin chains, and is one of the most prevalent monogenic diseases in clinical practice [1]. It is widely distributed at Mediterranean regions, Middle East, Indian subcontinent and Southeast Asia. According to the defective globin chains, thalassemia can be classified into α-, β-, δ-, and δβ-thalassemia, among which α-thalassemia and β-thalassemia are the most common and clinically important forms [2]. Globally, it is estimated that about 5% of the population carries α-thalassemia alleles and 1.5% of the population carries β-thalassemia alleles [3,4]. The clinical manifestations of thalassemia spans from asymptomatic carrier states to severe life-threatening conditions. Unlike thalassemia silent and minor which typically are asymptomatic, individuals with thalassemia intermedia present different levels of anemia at infancy, while patients with α-thalassemia major die in utero or shortly after birth, and patients with β-thalassemia major suffer from severe anemia and need lifelong transfusions and oral iron chelation therapy [3,5,6]. Due to the high incidence and absence of radical treatment, thalassemia has become a serious medical and public health problem worldwide.

In China, thalassemia is most frequent in 10 southern provinces, including Guangdong, Guangxi, Fujian, Jiangxi, Hunan, Hainan, Chongqing, Sichuan, Yunnan and Guizhou [7,8,9]. Epidemiological data estimates that about 30 million Chinese carry thalassemia variants, with about 300,000 suffering from thalassemia intermedia or major. Leading the fight against thalassemia, the Guangxi government has launched free preconception carrier screening and prenatal diagnosis followed by medical intervention, which are recognized as optimal strategies for the prevention and control of thalassemia currently. However, given that the premarital and prenatal programs are not mandatory in China, it is difficult to completely achieve the goal of thalassemia prevention and control only by these strategies. Therefore, universal neonatal thalassemia screening should be performed so that intervention therapy can be provided as early as possible, especially for the babies of couples who have not accessed thalassemia screening services. Despite the critical role, the progress of neonatal thalassemia screening lags far behind carrier screening and prenatal diagnosis strategies.

Conventionally, the methods used for screening and diagnosis of thalassemia include routine blood test, hemoglobin electrophoresis, and confirmatory genetic analysis. Individuals are initially subjected to routine blood test and hemoglobin assay to examine whether they have abnormal hematological results, such as reduced levels of mean corpuscular volume and mean corpuscular hemoglobin. The positive ones are further genetically analyzed to confirm their genotypes. Currently, most clinical laboratories use Gap-PCR and reverse dot blot (RDB) to detect 23 or 24 common variants of α-thalassemia and β-thalassemia, which account for more than 95% of the Chinese population [8,10,11]. Nevertheless, these methods mentioned above still have some limitations. First, thalassemia silent is ignored in the hematological test due to normal indices. Second, up to now more than 100 variants of α-thalassemia genes and 140 variants of β-thalassemia gene have been documented in the Chinese population (data from The Human Variome Project in China), and more novel variants are still booming. Most of them are missed by routine Gap-PCR, and RDB methods fail to detect most of these rare and complex variants, leading to missed diagnosis and misdiagnosis. Although the application of next-generation sequencing (NGS) in thalassemia detection expends the detection range of variants [12,13], the nature of short read sequencing limits its widespread clinical application. Recently, the comprehensive analysis of thalassemia alleles (CATSA) based on single molecule real-time (SMRT) sequencing technology is validated as a more accurate and comprehensive strategy to detect both common and rare variants of thalassemia genes, and shows great advantages over conventional genetic methods in carrier screening, prenatal diagnosis and newborn screening [14,15,16,17].

The “Guangxi Model” exemplifies a successful, integrated public health response to thalassemia and serves as a benchmark for other prevalent regions. Therefore, using Guangxi as an example, a prospective, multi-center neonatal thalassemia screening was performed by directly using SMRT, aiming to investigate the epidemiological features of thalassemia in this subpopulation. By leveraging this study, we not only characterized the genomic landscape of thalassemia in newborns, but also achieved early identification of newborns with thalassemia intermedia or major. These findings demonstrated the clinical utility of SMRT sequencing in neonatal thalassemia screening, providing evidence for the precision prevention and control of thalassemia in Guangxi.

## 2. Materials and Methods

### 2.1. Participants

Newborns were prospectively recruited from centers in Nanning, Beihai and Qinzhou. A total of 3671 newborns (Nanning: 1574; Beihai: 1148; Qinzhou: 949) were included. In total, 3667 dried blood spot (DBS) samples and 4 peripheral blood samples were collected in this study. Guardians of all enrolled neonates signed the informed consent form and volunteered to participate in thalassemia genetic screening.

### 2.2. DNA Extraction

An 8 mm punch of DBS sample or 200 μL of peripheral blood was collected. Genomic DNA was extracted using a magnetic bead-based method with a Nucleic Acid Extraction Kit (GenMagBio, Changzhou, China). A total of 20 ng of genomic DNA from DBS samples or 50 ng from peripheral blood samples was used for subsequent analysis.

### 2.3. SMRT Sequencing and Data Analysis

CATSA analysis was performed as described previously [17]. Briefly, genomic DNA was amplified by multiplex long PCR with primers encapsulating structural variant regions, single nucleotide variations (SNVs), as well as small insertions and deletions (InDels) in the *HBA1*, *HBA2*, *HBB*, *HBD* genes, and HS-40 deletion. For each amplified sample, a unique identifying barcode adaptor was added by a one-step end-repair and ligation reaction. The pooled pre-libraries were further utilized to generate SMRTbell libraries by using the Sequel Binding and Internal Ctrl Kit 3.0 (Pacific Biosciences, Menlo Park, CA, USA) and further sequenced on the PacBio Sequel II platform. The generated raw reads were converted to circular consensus sequencing (CCS) reads by CCS softwareversion 6.0.0 (Pacific Biosciences, Menlo Park, CA, USA), followed by alignment to human reference genome (hg38) for calling of SNVs, InDels, and structural variants. Pathogenicity of the variants was based on the hemoglobin variant databases including HbVar (https://globin.bx.psu.edu/hbvar/, accessed on 10 December 2024) and Ithanet (https://www.ithanet.eu, accessed on 10 December 2024).

### 2.4. Statistical Analysis

Data were analyzed using GraphPad Prism (version 10.1.2). To compare the carrier rates among sexes and regions, the chi-square (χ^2^) test was used and the statistical significance was set at *p* < 0.05 for two tails.

## 3. Results

### 3.1. Thalassemia Carrier Rates

In the study, a total of 3671 newborns were recruited for thalassemia screening by SMRT sequencing. Of them, 956 newborns were identified to carry at least one thalassemia variant, with a carrier rate of 26.04% (Table 1). In detail, the subgroup α-thalassemia was the most common with a carrier rate of 18.31% (672/3671), followed by β-thalassemia (6.21%, 228/3671), combined α/β-thalassemia (1.50%, 55/3671), and δ-thalassemia (0.03%, 1/3671).

Geographically, the carrier rate of Beihai (16.64%, 191/1148) was much lower than that of Nanning (30.37%, 478/1574) and Qinzhou (30.24%, 287/949) with statistically significant differences. Also, similar tendencies were seen in the subgroups of thalassemia. For the frequencies of thalassemia, no statistically significant difference was observed between females and males.

### 3.2. Frequency of Thalassemia Variants and Genotypes

**Figure 1:**
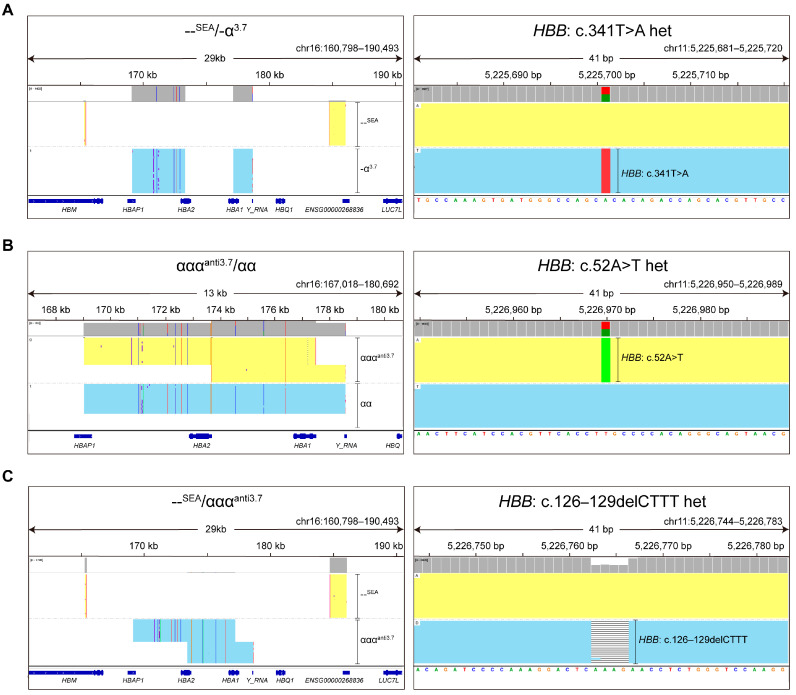
Integrative Genomics Viewer (IGV) plots of variants identified by SMRT. (**A**) IGV plots of --^SEA^/-α^3.7^ combined with heterozygous Hb New York. (**B**) IGV plots of heterozygous CD 17 (A>T) combined with α triplication (ααα^anti3.7^). (**C**) IGV plots of --^SEA^/ααα^anti3.7^ combined with CD 41/42 (-TTCT). Reads from the two alleles are indicated by different colors: yellow and blue.

Collectively, nine distinct types of clinically significant α-thalassemia variants were identified in the study, including five deletions and four non-deletions. Among them, --^SEA^ was the most frequent followed by six other hotspot variants that completely covered more than 99% of the carriers (Table 2). Additionally, two rare variants including -α^2.4^ and Init CD (ATG>A-G) were also detected. These variants combined into multiple types of genotypes in 672 α-thalassemia carriers (Table 3), among which --^SEA^/αα was the most prevalent accounting for 40.03% (269/672), while -α^3.7^/αα and α^WS^α/αα took the second (21.43%, 144/672) and third (12.35%, 83/672) places respectively. According to the genotypes, the carriers were classified into three groups: (1) 12 newborns (1.79%, 12/672) with hemoglobin H (Hb H) disease, all of whom were from Nanning and Qinzhou and none from Beihai; (2) 357 newborns (53.13%, 357/672) with α-thalassemia minor; (3) 303 newborns (45.09%, 303/672) with α-thalassemia silent. It was worth mentioning that seven α-thalassemia carriers coinherited other types of abnormal hemoglobinopathies, especially one Hb H disease case (Figure 1A). In addition, we also identified that five carriers with α-thalassemia silent or minor were combined with α triplication.

A total of 11 different β-thalassemia variants were found, all of which were point variants. Of them, CD 41/42 (-TTCT), CD 17 (A>T), and −28 (A>G) were the top three frequent variants (Table 2). Two rare variants of β-thalassemia were detected, including −50 (G>A) and IVS-II-5 (G>C). A total of 13 kinds of genotypes were detected among the 228 β-thalassemia carriers (Table 4), and all of them were heterozygotes. Among the heterozygotes, heterozygous CD41/42(-TTCT) was the most common (35.53%, 81/228), and heterozygous CD17(A>T) (24.12%, 55/228) as well as −28(A>G) (10.96%, 25/228) were less common. No homozygotes were found in this study. However, two participants were observed to carry a combination of a heterozygous CD 17(A>T) variant and αααanti3.7 (Figure 1B), which would enhance the imbalance between α and β globin chains, thus converting the phenotype from β-thalassemia minor to β-thalassemia intermedia. Additionally, we also detected one carrier who coinherited an abnormal Hb variant.

Among the newborns, 55 were identified to be carriers of both α and β thalassemia (Table 5) with a carrier rate of 1.50% (55/3671). They presented diverse genotypes, and more than 78.18% of them were caused by coinheritance of common α-globin genes deletions (--^SEA^/αα, -α^3.7^/αα or -α^4.2^/αα) with β-globin gene point variants. Interestingly, a newborn with combined α/β-thalassemia also carried α triplication (Figure 1C), which further complicated the newborn’s phenotype. Beyond those cases with prevalent thalassemia types, we also observed a newborn with δ-thalassemia caused by heterozygous *HBD*:c.-127T>C.

### 3.3. Detection of α-Globin Genes Structural Variants

Triplications of α-globin genes were detected in 37 newborns (Table 6), with a carrier rate of 1.01% (37/3671). They included 25 cases with ααα^anti3.7^ and 12 cases with ααα^anti4.2^. In the former group, four cases had combined α-thalassemia, two participants had combined β-thalassemia, as well as one participant coinherited with both α-thalassemia and β-thalassemia. Only one case was found to be combined with thalassemia in the latter group. In addition, we also observed eight cases with HKαα and two cases with antiHKαα.

### 3.4. Other Types of Hemoglobinopathies

A total of 34 participants were identified to be carriers of 11 types of abnormal hemoglobin variants (Table 6) presenting a carrier rate of 0.93% (34/3671). Hb Hekinan II had the highest incidence among the hemoglobin abnormalities accounting for 35.29% (12/34). Among them, eight participants were found to suffer from abnormal hemoglobin variants and thalassemia variants simultaneously.

## 4. Discussion

Thalassemia is an epidemic in Guangxi Province and has become a major public health concern there. Early and accurate screening and diagnosis play critical roles in thalassemia prevention and control. Herein, we prospectively applied SMRT to 3671 neonates from multiple centers in Guangxi for screening of thalassemia. By leveraging this study, we not only characterize the genomic landscape of thalassemia in newborns here, but also demonstrate the clinical utility of SMRT in neonatal thalassemia screening.

Since 2010, Guangxi has launched many thalassemia prevention and control programs, including free carrier screening for couples planning for pregnancy, confirmatory genetic analysis for positive ones, and prenatal diagnosis for high-risk families followed by medical intervention for fetuses with thalassemia major. Despite these efforts, several limitations persist in the current prevention and control strategies. On one hand, not all individuals at childbearing period actually perform carrier screening and prenatal diagnosis, leaving a risk of bearing affected offspring. On the other hand, current genetic methods show a limited detection range with only existing hotspot variants in the Chinese population, which may lead to misdiagnosis and missed diagnosis. Thus, neonatal thalassemia screening through a more comprehensive genetic analysis would be particularly important for the early identification and treatment of those babies. In recent years, SMRT sequencing has emerged as a robust alternative for thalassemia detection, offering great advantages over conventional PCR-based methods. First, SMRT sequencing demonstrates superior detection accuracy. A prospective multi-center study evaluating its clinical utility for identifying both α- and β-thalassemia genetic carrier status not only confirmed 100% concordance with conventional methods with no false negatives or false positives, but also exclusively identified 34 variants, all of which were validated by a combination of specifically designed PCR and Sanger sequencing [14]. Similarly, SMRT sequencing also achieved 100% accuracy in 278 fetuses from at-risk pregnancies. Meanwhile, PCR-based methods had one false positive and two false negatives [15]. Second, SMRT sequencing enables comprehensive detection of all globin variants in a single assay, whereas at least two separate PCR-based assays are required to cover deletion and nondeletion variants. Third, SMRT sequencing can directly determine the cis/trans configuration of two or more variants, facilitating confirmation of their parental origin. Therefore, this study prospectively performed a multi-center neonatal thalassemia screening in Guangxi by using SMRT and found that 0.38% (14/3671) of the newborns carried thalassemia intermedia, for whom long-term management, individualized treatment plans, and life guidance were recommended. Notably, genotypes of the two newborns with β-thalassemia intermedia were a combination of heterozygous β^CD17(A>T)^ with ααα^anti3.7^. Thus, 14.29% (2/14) of affected infants would be misdiagnosed by routine hotspot variants analysis. Collectively, these results underscore the clinical necessity of integrating SMRT sequencing-based neonatal thalassemia screening into prevention system. With the cost of library preparation and sequencing reagent reduces to approximately 20$ per sample, SMRT sequencing is increasingly feasible as a first-tier, population-based neonatal screening tool, particularly in regions with a high incidence of thalassemia [18].

The epidemiological data of thalassemia in Guangxi had been reported in many studies, with an estimated incidence ranging from 16.54% to 29.74% [19,20,21,22,23]. Our study showed some advantages compared to these studies. First, our study captured the genetic heterogeneity of thalassemia, because we directly performed SMRT for all recruited individuals. In addition to 16 common variants in the Chinese population, 20 rare variants also were identified, expanding the variant spectrum of globin genes in Guangxi. Importantly, several rare variants had critical clinical implications. For example, α triplications (such as ααα^anti3.7^ and ααα^anti4.2^) were considered to enhance the disease severity theoretically when combined with β-thalassemia variants. Considering the high frequency showed in our study, they were recommended to be integrated into routine clinical testing. However, most of the data in the past were based on analysis of hotspot variants for ones positive for hematological screening, thus the incidence of thalassemia may be underestimated in these studies. Second, previous studies mostly focused on adults, but provided little information about newborns. As we knew, our study presents the largest report, which systematically provides the epidemiological characteristics of thalassemia in this subpopulation of Guangxi.

We found that the genomic landscape of thalassemia exhibited sub-regional divergence. First, the carrier rate of thalassemia in newborns differed among sub-regions of Guangxi, with a lower rate in Beihai compared to Naning and Qinzhou, and none of thalassemia intermedia cases were from Beihai. This may be due to economics, demographic composition, and population migration. Second, --^SEA^ and CD 41/42 (-TTCT) were the most common variants for α-thalassemia and β-thalassemia, respectively, in Guangxi. However, these results differed from findings from Guizhou where -α^3.7^ and CD 17 (A>T) were the most common α-thalassemia and β-thalassemia variants [12], Hunan where -α^3.7^ and IVS-II-654 (C>T) were the most common ones [13], and the southern area of Hainan where –α^4.2^ was the most common α-thalassemia variant [24].

This study has several limitations. First, the SMRT sequencing assay was designed to target the variant sites associated with thalassemia on the *HBA1/2*, *HBB*, and *HBD* genes, as well as the HS-40 upstream regulatory element. While the detection range of SMRT sequencing assay could be expanded by incorporating additional primers pairs, to encompass more core genes and regulatory elements, such as *KLF1* [25], the cost inevitably increased. Thus, any expansion must balance enhanced diagnostic breadth against practical feasibility in large-scale screening programs. Second, long-term follow-up studies are warranted to monitor the growth, development, and hematologic parameters of the enrolled newborns. Such longitudinal data are essential for interrelated purposes: (1) evaluating the long-term clinical utility of neonatal thalassemia screening by SMRT sequencing in thalassemia prevention and control; and (2) refining genotype–phenotype correlations to support evidence-based disease stratification, thereby enhancing the accuracy and efficiency of carrier screening and prenatal diagnosis. Third, because this study exclusively focuses on newborns, its finding may not accurately reflect the actual burden of thalassemia in Guangxi.

## 5. Conclusions

In conclusion, this prospective, multi-center cohort study represents a large-scale molecular epidemiological survey of thalassemia in newborns of Guangxi by directly using SMRT. Further, this study provided evidence of SMRT sequencing as a comprehensive and accurate method for neonatal thalassemia screening. In the era of precision prevention and control, the results would provide a basis for formulating and refining evidence-based thalassemia prevention strategies in Guangxi.

## Figures and Tables

**Table 1:** Carrier rates of thalassemia in the newborns.

	NO.	Total Carrier Rate	α-thal Carrier Rate	β-thal Carrier Rate	Combined α/β-thal Carrier Rate	δ-thal Carrier Rate
Total	3671	956 (26.04%)	672 (18.31%)	228 (6.21%)	55 (1.50%)	1 (0.03%)
Region						
Nanning	1574	478 (30.37%)	332 (21.09%)	115 (7.31%)	30 (1.91%)	1 (0.06%)
Beihai	1148	191 (16.64%)	139 (12.11%)	48 (4.18%)	4 (0.35%)	0
Qinzhou	949	287 (30.24%)	201 (21.18%)	65 (6.85%)	21 (2.21%)	0
Gender						
Female	1700	459 (27.00%)	315 (18.53%)	123 (7.24%)	20 (1.18%)	1 (0.06%)
Male	1971	497 (25.22%)	357 (18.11%)	105 (5.33%)	35 (1.78%)	0

NO.: number of the cases; thal: thalassemia.

**Table 2:** Variants identified in the newborns.

Common Name	Hb Name	HGVS	Total		Nanning		Beihai		Qinzhou	
			NO.	Proportion	NO.	Proportion	NO.	Proportion	NO.	Proportion
**α-thal**										
--^SEA^	NA	NC_000016.10: g.165401_184701del	314	41.98%	150	40.11%	56	38.89%	108	46.96%
-α^3.7^	NA	NG_000006.1: g.34247_38050del	170	22.73%	81	21.66%	43	29.86%	46	20.00%
CD122 (CAC>CAG)	Hb Westmead (WS)	*HBA2*: c.369C>G	98	13.10%	56	14.97%	16	11.11%	26	11.30%
-α^4.2^	NA	NC_000016.10: g.169818_174075del	79	10.56%	36	9.63%	20	13.89%	23	10.00%
CD142 (TAA>CAA)	Hb Constant spring (CS)	*HBA2*: c.427T>C	69	9.22%	42	11.23%	5	3.47%	22	9.57%
CD 125 (CTG>CCG)	Hb Quong Sze (QS)	*HBA2*: c.377T>C	13	1.74%	7	1.87%	2	1.39%	4	1.74%
--^THAI^	NA	NC_000016.10: g.149863_183312del	2	0.27%	1	0.27%	0	0.00%	1	0.43%
**-α^2.4^**	NA	NG_000006.1: g.36859_39252del	2	0.27%	1	0.27%	1	0.69%	0	0.00%
**Init CD (ATG>A-G)**	NA	*HBA2*: c.2delT	1	0.13%	0	0.00%	1	0.69%	0	0.00%
**Total**			**748**	**100%**	**374**	**100%**	**144**	**100%**	**230**	**100%**
**α Structural Variants**										
**ααα^anti3.7^**	NA	NG_000006.1: g.34247_38050dup	25	53.19%	9	69.23%	5	29.41%	11	64.71%
**ααα^anti4.2^**	NA	NG_000006.1: g.(31957_31978)_(34525_34544)dup	12	25.53%	2	15.38%	7	41.18%	3	17.65%
**HKαα**	NA	NA	8	17.02%	2	15.38%	3	17.65%	3	17.65%
**antiHKαα**	NA	NA	2	4.26%	0	0.00%	2	11.76%	0	0.00%
**Total**			**47**	**100%**	**13**	**100%**	**17**	**100%**	**17**	**100%**
**β-thal**										
CD 41/42 (-TTCT)	NA	*HBB*: c.126-129delCTTT	104	36.75%	53	36.55%	18	34.62%	33	38.37%
CD 17 (A>T)	NA	*HBB*: c.52A>T	71	25.09%	39	26.90%	7	13.46%	25	29.07%
−28 (A>G)	NA	*HBB*: c.-78A>G	29	10.25%	10	6.90%	9	17.31%	10	11.63%
IVS-II-654 (C>T)	NA	*HBB*: c.316-197C>T	23	8.13%	11	7.59%	8	15.38%	4	4.65%
CD 71/72 (+A)	NA	*HBB*: c.217dupA	20	7.07%	11	7.59%	3	5.77%	6	6.98%
CD 26 (GAG>AAG)	Hb E	*HBB*: c.79G>A	16	5.65%	11	7.59%	1	1.92%	4	4.65%
IVS-I-1 (G>T)	NA	*HBB*: c.92+1G>T	8	2.83%	5	3.45%	1	1.92%	2	2.33%
**−50 (G>A)**	NA	*HBB*: c.-100G>A	7	2.47%	2	1.38%	3	5.77%	2	2.33%
CD 43 (G>T)	NA	*HBB*: c.130G>T	2	0.71%	1	0.69%	1	1.92%	0	0.00%
Cap +43/+40(-AAAC)	NA	*HBB*: c.-11_-8delAAAC	2	0.71%	1	0.69%	1	1.92%	0	0.00%
**IVS-II-5 (G>C)**	NA	*HBB*: c.315+5G>C	1	0.35%	1	0.69%	0	0.00%	0	0.00%
**Total**			**283**	**100%**	**145**	**100%**	**52**	**100%**	**86**	**100%**
**δ-thal**										
**−77(T>C)**	NA	*HBD*:c.- 127T>C	1	100%	1	100%	0	0	0	0
**Hb Variants**										
**CD 27 (GAG>GAT)**	Hb Hekinan II	*HBA1*: c.84G>T	14	40.00%	5	45.45%	3	30.00%	6	42.86%
**CD 113 (GTG>GAG)**	Hb New York	*HBB*: c.341T>A	5	14.29%	3	27.27%	1	10.00%	1	7.14%
**CD 13 (GCC>TCC)**	Hb Binyang	*HBA2*: c.40G>T	5	14.29%	2	18.18%	2	20.00%	1	7.14%
**CD 121 (GTG>ATG)**	Hb Owari	*HBA1*: c.364G>A	3	8.57%	0	0.00%	2	20.00%	1	0.00%
**CD 30 (GAG>CAG)**	Hb G-Honolulu	*HBA1*: c.91G>C	2	5.71%	0	0.00%	1	10.00%	1	0.00%
**CD 69 (GGT>AGT)**	Hb City of Hope	*HBB*: c.208G>A	1	2.86%	1	9.09%	0	0.00%	0	9.09%
**CD 22 (GAA>GCA)**	Hb G-Coushatta	*HBB*: c.68A>C	1	2.86%	0	0.00%	1	10.00%	0	0.00%
**CD 19 (AAC>AAA or AAG)**	Hb D-Ouled Rabah	*HBB*: c.60C>A|*HBB*: c.60C>G	1	2.86%	0	0.00%	0	0.00%	1	0.00%
**CD 56 (GGC>GAC)**	Hb J-Bangkok	*HBB*: c.170G>A	1	2.86%	0	0.00%	0	0.00%	1	0.00%
**CD 74 (GAC>CAC)**	Hb Q-Thailand	*HBA1*: c.223G>C	1	2.86%	0	0.00%	0	0.00%	1	0.00%
**CD 32 (ATG>ATA)**	Hb Amsterdam-A1	*HBA1*: c.99G>A	1	2.86%	0	0.00%	0	0.00%	1	0.00%
**Total**			**35**	**100%**	**11**	**100%**	**10**	**100%**	**14**	**100%**

Hb: hemoglobin; HGVS: Human Genome Variation Society; NO.: number of the cases; NA: not available; thal: thalassemia. Variants beyond the detection range of conventional screening methods (Gap-PCR and RDB) are presented in bold.

**Table 3:** The genotype distribution of α-thalassemia.

Categories	Genotypes	Total		Nanning		Beihai		Qinzhou	
		NO.	Carrier Rate	NO.	Carrier Rate	NO.	Carrier Rate	NO.	Carrier Rate
Hb H disease	--^SEA^/-α^3.7^	5	0.14%	2	0.13%	0	0	3	0.32%
	--^SEA^/α^WS^α	4	0.11%	2	0.13%	0	0	2	0.21%
	--^SEA^/-α^4.2^	1	0.03%	1	0.06%	0	0	0	0.00%
	--^SEA^/α^QS^α	1	0.03%	1	0.06%	0	0	0	0.00%
α-thal minor	--^SEA^/αα	269	7.33%	124	7.88%	52	4.53%	93	9.80%
	α^CS^α/αα	61	1.66%	37	2.35%	4	0.35%	20	2.11%
	α^QS^α/αα	10	0.27%	6	0.38%	2	0.17%	2	0.21%
	--^THAI^/αα	2	0.05%	1	0.06%	0	0.00%	1	0.11%
	-α^3.7^/ -α^3.7^	2	0.05%	1	0.06%	0	0.00%	1	0.11%
	-α^3.7^/α^CS^α	2	0.05%	1	0.06%	1	0.09%	0	0.00%
	-α^3.7^/α^WS^α	1	0.03%	0	0.00%	0	0.00%	1	0.11%
	-α^4.2^/α^WS^α	1	0.03%	0	0.00%	0	0.00%	1	0.11%
	-α^3.7^/-α^4.2^	1	0.03%	1	0.06%	0	0.00%	0	0.00%
	α^WS^α/α^WS^α	1	0.03%	1	0.06%	0	0.00%	0	0.00%
	α^WS^α/α^CS^α	1	0.03%	1	0.06%	0	0.00%	0	0.00%
α-thal silent	-α^3.7^/αα	144	3.92%	69	4.38%	41	3.57%	34	3.58%
	α^WS^α/αα	83	2.26%	47	2.99%	16	1.39%	20	2.11%
	-α^4.2^/αα	68	1.85%	30	1.91%	20	1.74%	18	1.90%
	-α^2.4^/αα	2	0.05%	1	0.06%	1	0.09%	0	0.00%
	α^Init CD (ATG>A-G)^α/αα	1	0.03%	0	0.00%	1	0.09%	0	0.00%
α-thal minor & α triplication	--^SEA^/ααα^anti3.7^	1	0.03%	1	0.06%	0	0.00%	0	0.00%
	--^SEA^/ααα^anti4.2^	1	0.03%	1	0.06%	0	0.00%	0	0.00%
α-thal silent & α triplication	-α^3.7^/ααα^anti3.7^	3	0.08%	1	0.06%	0	0.00%	2	0.21%
Hb H & Hb variant	--^SEA^/-α^3.7^ & β^New York^/β^N^	1	0.03%	1	0.06%	0	0.00%	0	0.00%
α-thal minor & Hb variant	--^SEA^/α^G-Honolulu^α	1	0.03%	0	0.00%	1	0.09%	0	0.00%
	--^SEA^/αα & β^J-Bangkok^/β^N^	1	0.03%	0	0.00%	0	0.00%	1	0.11%
	--^SEA^/α^Binyang^α	1	0.03%	1	0.06%	0	0.00%	0	0.00%
	--^SEA^/α^Hekinan II^α	1	0.03%	1	0.06%	0	0.00%	0	0.00%
α-thal silent & Hb variant	α^CS^α/αα & β^D-Ouled Rabah^/β^N^	1	0.03%	0	0.00%	0	0.00%	1	0.11%
	-α^4.2^/α^Q-Thailand^α	1	0.03%	0	0.00%	0	0.00%	1	0.11%

NO.: number of the cases; thal: thalassemia.

**Table 4:** The genotype distribution of β-thalassemia.

Categories	Genotypes	Total		Nanning		Beihai		Qinzhou	
		NO.	Carrier Rate	NO.	Carrier Rate	NO.	Carrier Rate	NO.	Carrier Rate
β-thal intermediate	ααα^anti3.7^/αα & β^CD17(A>T)^/β^N^	2	0.05%	1	0.06%	0	0.00%	1	0.11%
β-thal minor	β^CD 41/42(-TTCT)^/β^N^	81	2.21%	41	2.60%	17	1.48%	23	2.42%
	β^CD 17(A>T)^/β^N^	55	1.50%	29	1.84%	6	0.52%	20	2.11%
	β^−28(A>G)^/β^N^	25	0.68%	8	0.51%	8	0.70%	9	0.95%
	β^IVS-II-654(C>T)^/β^N^	20	0.54%	10	0.64%	7	0.61%	3	0.32%
	β^CD 71/72(+A)^/β^N^	17	0.46%	9	0.57%	3	0.26%	5	0.53%
	β^CD 26 (GAG>AAG)^/β^N^	10	0.27%	7	0.44%	1	0.09%	2	0.21%
	β^−50(G>A)^/β^N^	6	0.16%	2	0.13%	3	0.26%	1	0.11%
	β^IVS-I-1(G>T)^/β^N^	6	0.16%	4	0.25%	1	0.09%	1	0.11%
	β^CD 43(G>T)^/β^N^	2	0.05%	1	0.06%	1	0.09%	0	0.00%
	β^Cap +43/+40(-AAAC)^/β^N^	2	0.05%	1	0.06%	1	0.09%	0	0.00%
	β^IVS-II-5(G>C)^/β^N^	1	0.03%	1	0.06%	0	0.00%	0	0.00%
β-thal minor & Hb variant	β^CD17(A>T)^/β^Binyang^	1	0.03%	1	0.06%	0	0.00%	0	0.00%

NO.: number of the cases; thal: thalassemia.

**Table 5:** The genotype distribution of combined α/β-thalassemia.

Categories	Genotypes	Total		Nanning		Beihai		Qinzhou	
		NO.	Carrier Rate	NO.	Carrier Rate	NO.	Carrier Rate	NO.	Carrier Rate
α-thal minor & β-thal minor	--^SEA^/αα & β^CD 41/42 (-TTCT)^/β^N^	9	0.25%	6	0.38%	1	0.09%	2	0.21%
	--^SEA^/αα & β^CD 17 (A>T)^/β^N^	8	0.22%	6	0.38%	0	0.00%	2	0.21%
	--^SEA^/αα & β^−28 (A>G)^/β^N^	3	0.08%	1	0.06%	1	0.09%	1	0.11%
	α^CS^α/αα & β^CD 41/42 (-TTCT)^/β^N^	3	0.08%	2	0.13%	0	0.00%	1	0.11%
	--^SEA^/αα & β^IVS-II-654 (C>T)^/β^N^	2	0.05%	0	0.00%	1	0.09%	1	0.11%
	--^SEA^/αα & β^CD 26 (GAG>AAG)^/β^N^	2	0.05%	2	0.13%	0	0.00%	0	0.00%
	--^SEA^/αα & β^−50 (G>A)^/β^N^	1	0.03%	0	0.00%	0	0.00%	1	0.11%
	--^SEA^/αα & β^IVS-I-1(G>T)^/β^N^	1	0.03%	0	0.00%	0	0.00%	1	0.11%
	α^QS^α/αα & β^CD 26 (GAG>AAG)^/β^N^	1	0.03%	0	0.00%	0	0.00%	1	0.11%
	α^QS^α/αα & β^CD 17 (A>T)^/β^N^	1	0.03%	0	0.00%	0	0.00%	1	0.11%
	α^CS^α/αα & β^CD 17 (A>T)^/β^N^	1	0.03%	1	0.06%	0	0.00%	0	0.00%
α-thal silent & β-thal minor	-α^3.7^/αα & β^CD41/42(-TTCT)^/β^N^	5	0.14%	2	0.13%	0	0.00%	3	0.32%
	α^WS^α/αα & β^CD41/42(-TTCT)^/β^N^	3	0.08%	1	0.06%	0	0.00%	2	0.21%
	-α^4.2^/αα & β^CD26 (GAG>AAG)^/β^N^	2	0.05%	1	0.06%	0	0.00%	1	0.11%
	-α^4.2^/αα & β^CD41/42(-TTCT)^/β^N^	2	0.05%	1	0.06%	0	0.00%	1	0.11%
	-α^4.2^/αα & β^CD17(A>T)^/β^N^	2	0.05%	1	0.06%	0	0.00%	1	0.11%
	-α^3.7^/αα & β^CD71/72(+A)^/β^N^	1	0.03%	0	0.00%	0	0.00%	1	0.11%
	-α^3.7^/αα & β^CD17(A>T)^/β^N^	1	0.03%	0	0.00%	1	0.09%	0	0.00%
	-α^4.2^/αα & β^CD 71/72 (+A)^/β^N^	1	0.03%	1	0.06%	0	0.00%	0	0.00%
	-α^3.7^/αα & β^IVS-I-1 (G>T)^/β^N^	1	0.03%	1	0.06%	0	0.00%	0	0.00%
	-α^3.7^/αα & β^CD 26 (GAG>AAG)^/β^N^	1	0.03%	1	0.06%	0	0.00%	0	0.00%
	α^WS^α/αα & β^IVS-II-654 (C>T)^/β^N^	1	0.03%	1	0.06%	0	0.00%	0	0.00%
	α^WS^α/αα & β^CD 71/72 (+A)^/β^N^	1	0.03%	1	0.06%	0	0.00%	0	0.00%
	α^WS^α/αα & β^−28 (A>G)^/β^N^	1	0.03%	1	0.06%	0	0.00%	0	0.00%
α-thal silent & β-thal minor & α triplication	--^SEA^/ααα^anti3.7^ & β^CD41/42(-TTCT)^/β^N^	1	0.03%	0	0.00%	0	0.00%	1	0.11%

NO.: number of the cases; thal: thalassemia.

**Table 6:** The genotype distribution of structural variants and abnormal hemoglobin variants.

Genotypes	Total		Nanning		Beihai		Qinzhou	
	NO.	Carrier Rate	NO.	Carrier Rate	NO.	Carrier Rate	NO.	Carrier Rate
Structural Variants with or without Thalassemia								
αα/ααα^anti3.7^	18	0.49%	6	0.38%	5	0.44%	7	0.74%
-α^3.7^/ααα^anti3.7^	3	0.08%	1	0.06%	0	0.00%	2	0.21%
--^SEA^/ααα^anti3.7^	1	0.03%	1	0.06%	0	0.00%	0	0.00%
ααα^anti3.7^/αα & β^CD17(A>T)^/β^N^	2	0.05%	1	0.06%	0	0.00%	1	0.11%
--^SEA^/ααα^anti3.7^ & β^CD41/42(-TTCT)^/β^N^	1	0.03%	0	0.00%	0	0.00%	1	0.11%
ααα^anti4.2^/αα	11	0.30%	1	0.06%	7	0.61%	3	0.32%
--^SEA^/ααα^anti4.2^	1	0.03%	1	0.06%	0	0.00%	0	0.00%
HKαα/αα	8	0.22%	2	0.13%	3	0.26%	3	0.32%
antiHKαα/αα	2	0.05%	0	0.00%	2	0.17%	0	0.00%
Hemoglobin Variants with or without Thalassemia								
α^Hekinan II^α/αα	12	0.33%	4	0.25%	3	0.26%	5	0.53%
β^New York^/β^N^	4	0.11%	2	0.13%	1	0.09%	1	0.11%
α^Owari^α/αα	3	0.08%	0	0.00%	2	0.17%	1	0.11%
α^Binyang^α/αα	3	0.08%	0	0.00%	2	0.17%	1	0.11%
--^SEA^/α^Binyang^α	1	0.03%	1	0.06%	0	0.00%	0	0.00%
α^Binyang^α/αα & β^CD17(A>T)^/β^N^	1	0.03%	1	0.06%	0	0.00%	0	0.00%
β^City of Hope^/β^N^	1	0.03%	1	0.06%	0	0.00%	0	0.00%
α^G-Honolulu^α/αα	1	0.03%	0	0.00%	0	0.00%	1	0.11%
--^SEA^/α^G-Honolulu^α	1	0.03%	0	0.00%	1	0.09%	0	0.00%
β^G-Coushatta^/β^N^	1	0.03%	0	0.00%	1	0.09%	0	0.00%
α^Hekinan II^α/α^Amsterdam-A1^α	1	0.03%	0	0.00%	0	0.00%	1	0.11%
--^SEA^/αα & β^J-Bangkok^/β^N^	1	0.03%	0	0.00%	0	0.00%	1	0.11%
--^SEA^/α^Hekinan II^α	1	0.03%	1	0.06%	0	0.00%	0	0.00%
--^SEA^/-α^3.7^& β^New York^/β^N^	1	0.03%	1	0.06%	0	0.00%	0	0.00%
-α^4.2^/α^Q-Thailand^α	1	0.03%	0	0.00%	0	0.00%	1	0.11%
α^CS^α/αα & β^D-Ouled Rabah^/β^N^	1	0.03%	0	0.00%	0	0.00%	1	0.11%

NO.: number of the cases.

## Data Availability

Data supporting this work are available upon request.
